# Enhancing Spatial Memory: Anxiolytic and Antidepressant Effects of *Tapinanthus dodoneifolius* (DC) Danser in Mice

**DOI:** 10.1155/2014/974308

**Published:** 2014-02-05

**Authors:** Foyet Harquin Simplice, Tsala David Emery, Ngatanko Abaissou Hervé Hervé

**Affiliations:** ^1^Department of Biological Sciences, Faculty of Science, University of Maroua, P.O. Box 46, Maroua, Cameroon; ^2^Higher Teachers' Training College, University of Maroua, P.O. Box 55, Maroua, Cameroon

## Abstract

We evaluated the anxiolytic and antidepressant effects of the aqueous extract of the bark of *Tapinanthus dodoneifolius* (TAE) (Danser) (25, 50, and 100 mg/kg), using open field, elevated plus maze, and forced swimming tests. Effect of TAE was compared to standard drugs diazepam (2 mg/kg) and imipramine (10 mg/kg). Additionally, the same doses of TAE were evaluated on rat's memory using Y-maze task. Results showed a significant (*P* < 0.05; 100 mg/kg) increase in the percentage of open arm entry and the time spent in the open arms in the elevated plus maze, suggesting an anxiolytic activity of the extract. In a dose-dependant manner, TAE at 25 mg/kg significantly (*P* < 0.05) decreased the number of lines crossed and the rearing behavior in the open field test, suggesting its possible sedative activity. In the forced swimming test, the immobility time of the animal was significantly reduced (*P* < 0.05) by TAE (100 mg/kg), compared to control, and this effect was quite comparable to that of imipramine. In the Y-maze paradigm, TAE at 50 mg/kg caused a significant increase in the spontaneous alternations but with a significant decrease in exploratory behavioral pattern. Taking these results together, TAE improved the spatial memory and showed anxiolytic, antidepressant, and sedative activities. The present results support the anxiolytic and antidepressant activities of TAE and, to our knowledge, for the first time, demonstrate its enhancing effect on memory.

## 1. Introduction

One in four people in the world is affected by mental or neurological disorders at some point in their live. About 450 million people currently suffer from such conditions, placing mental disorders among the leading causes of ill-health and disability worldwide. According to the World Health Organization (WHO), the vast majority of countries allocate less than 2% of their health budgets to mental health, leading to a treatment gap of more than 75% in many low and middle income countries [1]. The poor often bear the greater burden of mental disorders, both in terms of the risk in having a mental disorder and the lack of access to treatment. Constant exposure to severely stressful events, dangerous living conditions, exploitation, and poor health in general contribute to the greater vulnerability of the population. The lack of access to affordable treatment makes the course of the illness more severe and debilitating, leading to a vicious circle of poverty and mental health disorders that is rarely broken [2].

Often referred to as the common cold of mental disorders, both anxiety and depression are debilitating conditions that greatly impair psychological, social, and emotional well-being. They represent the most frequent psychiatric conditions. More than 20% of the adult population suffer from these conditions at sometime during their life [3]. Anxiety disorders are common mental diseases of the central nervous system (CNS) that include heterogeneous phenomena such as panic disorder, phobias, obsessive-convulsive disorders, generalized anxiety disorders, and posttraumatic stress disorders [4] while depressive disorders are illnesses that involve the body, mood, and thought. Anxiety and depressive disorders have many causes: on one hand those related to environment, mode of life, modernization of habits, industrialization, worries, stressing professional situations, and economical and financial crisis. On the other hand, we have intrinsic causes that can be genetic, some diseases like cardiac and respiratory insufficiencies and malfunctioning of the thyroid gland [5].

For many years, anxiety and depression were considered as two different mental diseases, with the benzodiazepines used as drugs of choice for acute anxiety states and the amine uptake inhibitors to treat depression. Meanwhile the clinicians seem to have become less sure of the original mutually exclusive classification of the two diseases, with margin between anxiety and depression becoming blurred [4, 6, 7]. In the treatment of anxiety disorders, the benzodiazepines are now slowly replaced by antidepressants, which are not only efficacious in depression but also in acute and long-term treatment for major anxiety disorders [8]. The different drugs used act on GABA receptors (GABA_A_) as agonist at the receptors, increasing calcium influx. In a general manner, GABA acts to reduce the firing of the dopaminergic neurons. They also act on serotonergic systems [9]. However almost all pharmacological treatments used to diminish these disorders have adverse side effects including sedation, sleep disturbance, and sexual dysfunctioning and are not accessible to all due to poverty. There is a great interest in fundamental and clinical research to explore new therapeutic targets and new molecules acting on the central nervous system. At the same time, there is a growing interest of people across the country to the use of alternative medicine, particularly in the acute phase of neurological disorders. However, the evidence to recommend the use of herbal medicines in the treatment of these illnesses is still insufficient [10].

There are reports about the popular use of *Tapinanthus dodoneifolius* (DC) Danser (Loranthaceae) also called *Agelanthus dodoneifolius* (Polh and Wiens ) in treating hypertension [11] and malaria [12]. In the Far North region of Cameroon, it is used in the treatment of diabetes and cancer. During an ethnobotanical survey in this region we also found that the stem bark of *T. dodoneifolius* was systematically used by more than two thirds of traditional healers in the treatment of epilepsy. These observations indicate that this plant may certainly have some therapeutic effects on the CNS. Nevertheless, there is no scientific evidence about potential effects of *Tapinanthus dodoneifolius* on the neurological disorders. The main objective of the present study was then to evaluate the neuroprotective effects and memory improvement properties of TAE on animal models of anxiety and depression.

## 2. Material and Methods

### 2.1. Experimental Animals

Animals were adult albino Swiss male mice (12 weeks, 27 ± 2 g). They were purchased from LANAVET (Laboratoire National Vétérinaire, Garoua) and housed in groups of 5 in polypropylene cages with wood shavings as bedding, under a natural 12 h/12 h light/dark cycle (lights on at 7:00 a.m.) and controlled temperature (23 ± 2°C). The animals had free access to water and food, with the exception of 1 h before and during the experiments. The animals were not specifically handled prior to the experiments, with the exception of handling during necessary animal care (cleaning the cages) and drug administration (weighing, tail marking, and drug administration).

### 2.2. Plant Material

The stem bark of *Tapinanthus dodoneifolius* used in our experiments was collected in October 2012, from Mokon in the Far North region of Cameroon and identified by Mrs. Hamawa Youkouda, Agroforestrist, and Froumsia Moksia, Botanists, at the Department of Agriculture, Livestock and By-products of the Higher Institute of Sahel (ISS) of the University of Maroua. This was later authenticated at the National School of Fauna, Garoua, where the Voucher specimen already existed under the number HEFGN 7406.

### 2.3. Preparation of Aqueous Extract

The stem bark of the plant was chopped into small pieces and shade dried in the laboratory at room temperature for three weeks until all the water evaporate. One hundred gram of dried, ground material was extracted in 1000 mL of boiled distilled water and allowed to infuse for 60 minutes. The extract was later filtered using a Whatman GF/C (90 mm⌀) paper and evaporated to dryness in an oven (50°C). The yield of the extract was 3.6 w%.

### 2.4. Drugs

TAE (25, 50, and 100 mg/kg), diazepam (2 mg/kg; Roche, France), and imipramine (10 mg/kg, SIGMA, Italy) as well as vehicle (10 mL/kg) were administered intraperitoneally (i.p.). TAE dose was set based on a preliminary ethnopharmacological survey, as administered to human being.

### 2.5. Phytochemical Studies

Preliminary phytochemical screening was carried out according to classic laboratory methods as described by Harborne [13].

### 2.6. Behavioral Tests

In this study, the mice were assigned to independent groups (5 in each group), except in the Y-maze test where there were 6 animals per group. Each animal was tested in only one behavioral test. The mice were habituated to the experimental room 1 day before the tests. The procedures used in the present study were in accordance with the guide for the care and use of laboratory animals Eighth Edition (Copyright 2011 by the National Academy of Sciences). The experiments were performed in the morning (8–12 h), and the illumination level was 200 lux in the experimental room.

#### 2.6.1. Elevated Plus-Maze Test (EPM)

The elevated plus-maze apparatus consists of four arms (25 × 5 cm) elevated 40 cm above the floor, with each arm positioned at 90° relative to the adjacent arms. Open and closed arms were connected via a central area (5 × 5 cm) to form a plus sign. Mice were randomly assigned to experimental groups: negative control (normal saline 10 mL/kg, i.p.), positive control (diazepam 2.0 mg/kg, i.p.), and TAE (25, 50, and 100 mg/kg, i.p.). Testing commenced 30 minutes after administration of drug by placing a mouse on the central platform of the maze, facing an open arm. The number of entries into and the time spent on each of the two types of arms were recorded during 5 minutes [14, 15]. We also recorded in the apparatus behavioral parameters such as head-dips (dipping the head below the open arm of the EPM, with all four paws on an open arm), stretch attend postures (frequency with which the animal demonstrated forward elongation of the head and shoulders followed by retraction to the original position), numbers of grooming (duration of time the animal spent licking or scratching itself while stationary) and rearing (frequency with which the mice stood on their hind legs in the maze), and time in the central platform. The plus-maze was thoroughly cleaned with 70% ethanol between tests.

#### 2.6.2. Open Field Test (OFT)

The open field arena was made of white polywood (72 cm × 72 cm × 36 cm) and divided into twelve squares of equal areas. Mice were carried to the test room in their home cages and were handled by the base of their tails at all times. The five groups of animals received the various treatments as described above 30 minutes prior to the test. Each animal was placed into the center of the open field and allowed to explore the apparatus for 5 minutes. After the 5 minutes test, mice were returned in their home cages and the open field cleaned with 70% ethanol and permitted to dry between tests. The observed parameters were ambulations (the number of squares crossed with all four paws), numbers of grooming and rearing, and time spent at the center and at the border if the arena [16].

#### 2.6.3. Forced Swimming Test (FST)

The most widely used pharmacological model for assessing antidepressant activity [17] is the FST. The apparatus consisted of a transparent glass cylinder (30 cm high × 20 cm diameter) filled to a 20 cm depth with water maintained at 25 ± 2°C. In the pretest, mice were placed in the cylinder for 15 min, 24 h prior to the 5-min swimming test. Animals were distributed 30 minutes prior to the swimming test as in the EPM test, but diazepam was replaced by imipramine at 10 mg/kg. For the 6-minutes swimming test, the following behavioral responses were recorded by a trained observer during the last four minutes: swimming time, defined as movement time throughout the swim chamber, which included crossing into another quadrant, and immobility time considered when the mouse made no further attempts to escape except the movements necessary to keep its head above the water. Increases in active responses, such as climbing or swimming, and reduction in immobility, were considered as behavioral profiles consistent with an antidepressant-like action [18].

#### 2.6.4. Y-Maze Test

Y-maze test is widely used to assess exploratory behaviors, learning, and memory function in rodents [19, 20]. The Y-maze apparatus consisted of three identical arms (33 × 11 × 12 cm each) in which the arms are symmetrically separated at 120°. Animals were randomly divided into five groups of 5 or 6 animals: negative control (10 mL/kg of normal saline); TAE (25, 50, and 100 mg/kg); and positive control (diazepam 2.0 mg/kg). Because we were interested in the cognitive and not in the neuroendocrine changes induced by running-wheel activity, we tried to design our experiment in such a way that stress levels are not minimized. Y-maze learning can also be stressful to the mice because of the new environment. No habituation session was undertaken, and by so doing, stress was associated with this task due to the fact that the mice were not allowed to voluntarily enter the apparatus but were placed by the experimenter on an arm of the apparatus. Nonhabituated mice were gently placed at the end of one arm and were allowed to freely explore the Y-maze during 8 min. The number of arm visits; sequence of arm visits; rearing frequency; grooming frequency; and the number of lines crossed were recorded for each mouse by a trained observer. The maze arms were cleaned with 70% ethanol between tasks to remove residual odor. An actual alternation was defined as entries into all three arms consecutively. The number of maximum spontaneous alternation behaviors was then calculated by subtracting two units from the total number of arms entry, and the percent of spontaneous alternation was calculated as (actual alternations/maximum alternations) × 100 [21, 22]. Spontaneous alternation behavior is considered to reflect spatial working memory, which is a form of short-term memory while the total number of arm entries was considered to reflect spontaneous locomotor activity.

### 2.7. Statistical Analysis

Data were presented as mean ± SEM values. One way ANOVA followed by Newman-Keuls Multiple Comparison Test was performed using GraphPad Prism version 5.00 for Windows, GraphPad Software, San Diego California USA, http://www.graphpad.com/. A probability level of 0.05 or less was accepted as significant. Pearson's correlation coefficient and regression analysis were used to evaluate the connection between the working memory errors and some parameters like locomotion, grooming and rearing in the Y-maze test.

## 3. Results

### 3.1. Chemical Components Present in the Aqueous Stem Extract of *Tapinanthus dodoneifolius*

The phytochemical screening revealed that TAE contains proteins, alkaloids, glycosides, saponins, tannins, phenols, and flavonoids.

### 3.2. Anxiolytic Activity

#### 3.2.1. Entries in Open and Closed Arms

**Figure 1:**
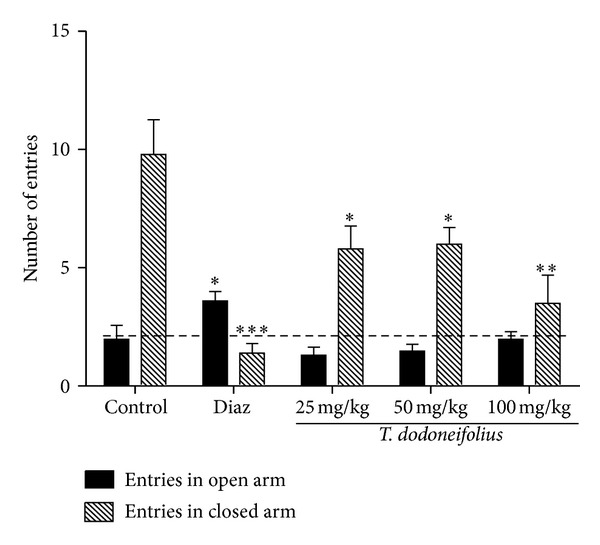
Effects of TAE or diazepam (Diaz) on the number of entries in the EPM. Animals were treated with the TAE (25, 50, or 100 mg/kg, i.p.), normal saline, or diazepam (2 mg/kg, i.p.). Each column represents the mean ± SEM of 5 animals. Data analysis was performed using Newman-Keuls Multiple Comparison Test, *P < 0.05; **P < 0.01; ***P < 0.001, significantly different from saline-treated animals.

In the EPM, TAE at the doses used significantly (*F*(3.28) = 5.30, *P* < 0.010) reduced the number of entries in closed arms in a dose-dependent manner, as compared to the control, with the higher activity at the higher dose, that is, 100 mg/kg (3.50 ± 2.06 versus 9.80 ± 2.92 for the control group). We also observe that diazepam brought about a highly significant (*F*(5.31) = 30.67, *P* < 0.0006) decrease in the number of entries in closed arms and an increase in the open arms (Figure 1).

#### 3.2.2. Time Spent in Open and Closed Arms

**Figure 2:**
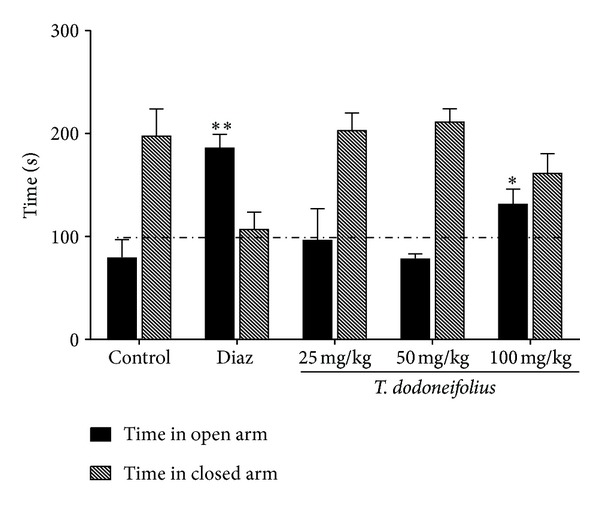
Effects of TAE or diazepam (Diaz) on the time spent in the different arms of the EPM. Animals were acutely treated with TAE (25, 50, or 100 mg/kg, i.p.) or normal saline. Diazepam was given only once as reference drug (2 mg/kg, i.p.). Each column represents the mean ± SEM of 5 animals. Data analysis was performed using Newman-Keuls Multiple Comparison Test, *P < 0.05, **p < 0.01, significantly different from saline-treated animals.

Figure 2 shows that in the EPM animals of the control group spent more time in the closed arm. When treated with the plant extract at the doses of 25 and 50 mg/kg, no remarkable changes were noticed. The highest dose of TAE (100 mg/kg) significantly increased (*F*  (5.31) = 7.30, *P* < 0.030) the time spent by the animals in the open arm compared to the control, with a concomitant decrease of the time spent at the closed arm. This was an index of the anxiolytic effect of the extract at this dose. Diazepam as the reference drug also exhibited this pharmacological activity with a more important effect.

#### 3.2.3. Rearing, Grooming, and Head Dipping Activities in the EPM

**Figure 3:**
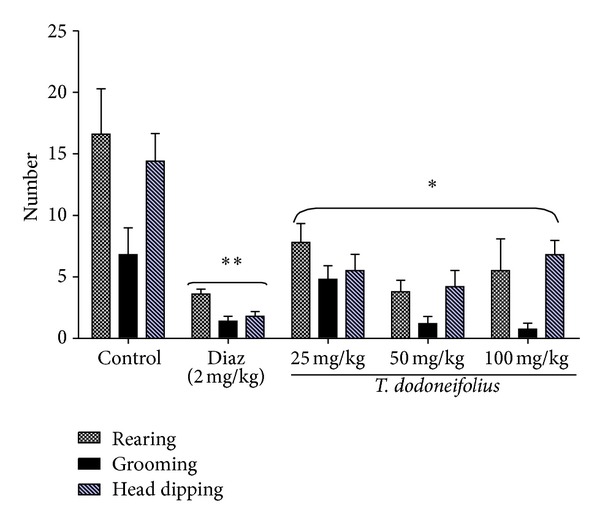
Effects of TAE or diazepam (Diaz) on rearing, grooming, and head dipping in the EPM. Animals were acutely treated with TAE (25, 50, or 100 mg/kg, i.p.) normal saline, or diazepam (2 mg/kg, i.p.). Each column represents the mean ± S.E.M. of 5 animals. Data analysis was performed using Newman-Keuls Multiple Comparison Test, *P < 0.05, **P < 0.01, significantly different from saline-treated animals.

As shown in Figure 3, the rearing, grooming, and head dipping behaviors were high in control animals in the EPM. But when the animals were pretreated with the different doses of TAE, the number of grooming significantly (*F*(3.28) = 4.62, *P* < 0.02) decreased in a dose-dependent manner with the highest effect obtained at the dose of 100 mg/kg. Interestingly, the inhibitory activity of the extract on the grooming behavior was more pronounced than that of diazepam (2 mg/kg), the reference drug. Similarly, the rearing which is a form of vertical locomotor activity also significantly (*F*(3.28) = 5.70, *P* < 0.009) decreased with the maximum effect at the dose of 50 mg/kg. The head dipping behavior in the elevated plus maze test was also significantly (*F*(3.28) = 8.39, *P* < 0.002) inhibited by the pretreatment of the animal with the extract (Figure 3).

#### 3.2.4. Ambulatory Activity in the OFT

**Figure 4:**
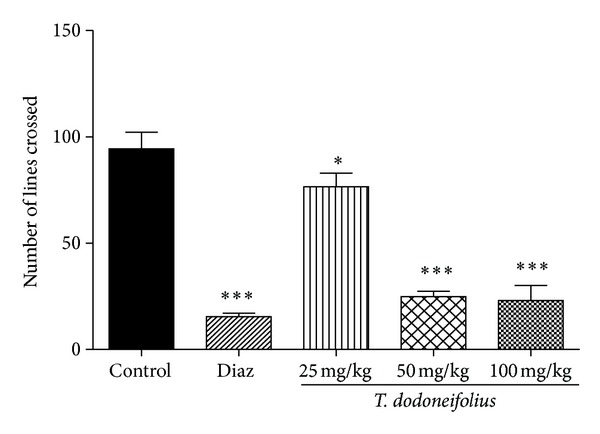
Effects of TAE or diazepam (Diaz) on the number of lines crossed by mice in the OFT. Animals were acutely treated with the extract (25, 50, or 100 mg/kg, i.p.), normal saline, or diazepam (2 mg/kg, i.p.). Each column represents the mean ± S.E.M. of 5 animals. Data analysis was performed using Newman-Keuls Multiple Comparison Test, *P < 0.05; ***P < 0.001, significantly different from saline-treated animals.

When placed in the open field arena, the control animals exhibited ambulatory activities marked by the number of lines crossed (94.40 ± 17.35). With the TAE pretreatment, the number of lines crossed severely dropped to 24 ± 3.4 and 23 ± 9.7, respectively, at the doses of 50 and 100 mg/kg (Figure 4). However, the inhibitory effect of diazepam on the locomotion was more pronounced than that of the plant extract.

#### 3.2.5. Time Spent at the Center and at the Border of the Arena in the OFT

The saline-treated animals passed all the time at the border of the field, spending about 4 min and 66 S of the 5 min test session at that zone. With the TAE treatment, a nonsignificant decrease in the time spent at the border of the maze was noticed (results not shown).

### 3.3. Effects of TAE in the FST

**Figure 5:**
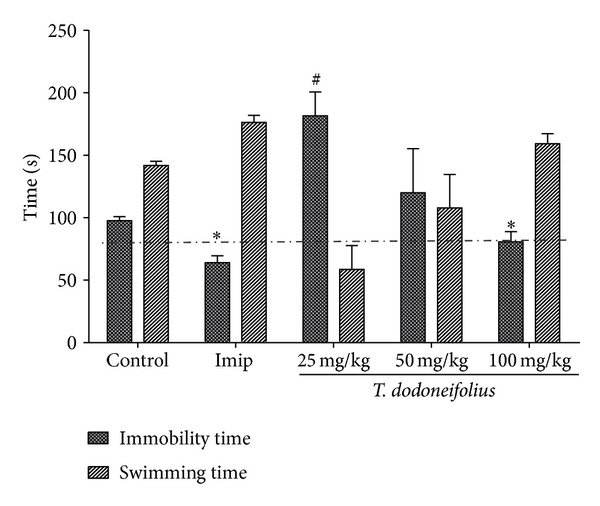
Effects of TAE or imipramine (Imip) on the FST in mice. Animals were acutely treated with TAE (25, 50, or 100 mg/kg, i.p.), normal saline, or imipramine (10 mg/kg, i.p.). Each column represents the mean ± S.E.M. of 5 animals. Data analysis was performed using Newman-Keuls Multiple Comparison Test, *P < 0.05, significantly different from saline-treated animals.

The results of this test revealed that the swimming time of the animals treated with TAE increased in a dose-dependant manner. However, the significant response was obtained only at the highest dose (100 mg/kg, (*F*(5.31) = 10.63, *P* < 0.011) as compared to the control group. The immobility time increased with the dose of 25 mg/kg and drops with doses of 50 mg/kg and 100 mg/kg. The standard drug in this test, imipramine, also decreased significantly the immobility time (Figure 5).

### 3.4. Effects of TAE in the Y-Maze Test

#### 3.4.1. Spontaneous Alternation

**Figure 6:**
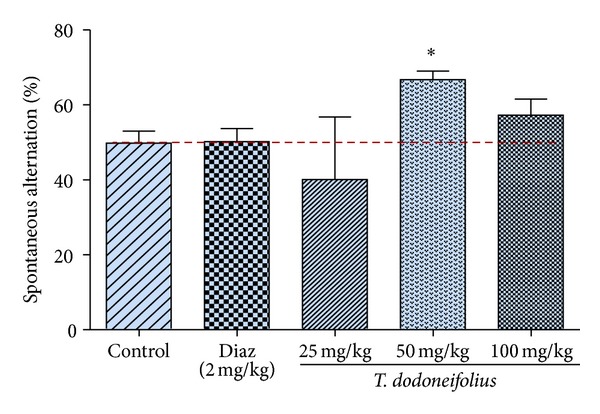
Effect of TAE and diazepam (Diaz) on the spontaneous alternation percentage in Y-maze task. Experiments were performed 30 min after single administration of the extract. Each column represents mean ± S.E.M. of 6 animals. Data analysis was performed using one way ANOVA followed by Newman-Keuls Multiple Comparison Test, *P < 0.05, significantly different from saline-treated animals.

After a single administration of TAE, there was an increase in the percentage of spontaneous alternation in animals treated with the intermediate dose (66.71% ± 9.51; 50 mg/kg) of the plant extract, when compared to control group (49.80% ± 8.42), suggesting effects on short-term memory. This increase in the percentage of spontaneous alternations was significant (*F*(2.92) = 1.93, *P* < 0.05) (Figure 6).

#### 3.4.2. Grooming, Rearing, and Lines Crossed

**Figure 7:**
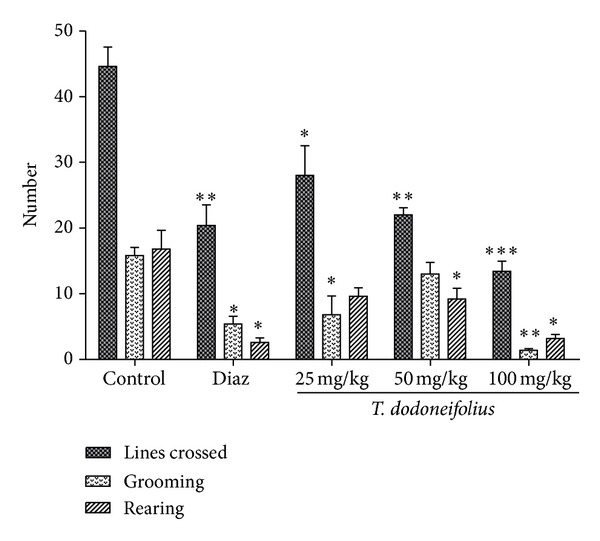
Effects of TAE and diazepam on the stress parameters in the Y-maze test. Animals were acutely treated with TAE (25, 50, or 100 mg/kg, i.p.), distilled water, or diazepam (10 mg/kg, i.p.). Each column represents the mean ± S.E.M. of 5-6 animals. Data analysis was performed using Newman-Keuls Multiple Comparison Test, *P < 0.05, **P < 0.01, ***P < 0.001, significantly different from saline-treated animals.

Figure 7 represents three stress parameters investigated in the Y-maze. The number of lines crossed in the treated animals decreased in a dose-dependent manner as compared to the saline treated animal (control). The effect obtained at a dose of 50 mg/kg was comparable to that of the standard drug diazepam (2 mg/kg). TAE also significantly reduced the grooming (*F*(3.23) = 12.73, *P* < 0.0002) and the rearing (*F*(3.23) = 9.69, *P* < 0.0007) behaviors of animals in this task.

#### 3.4.3. Correlation between Memory and Stress Parameters in the Y-Maze

**Figure 8:**
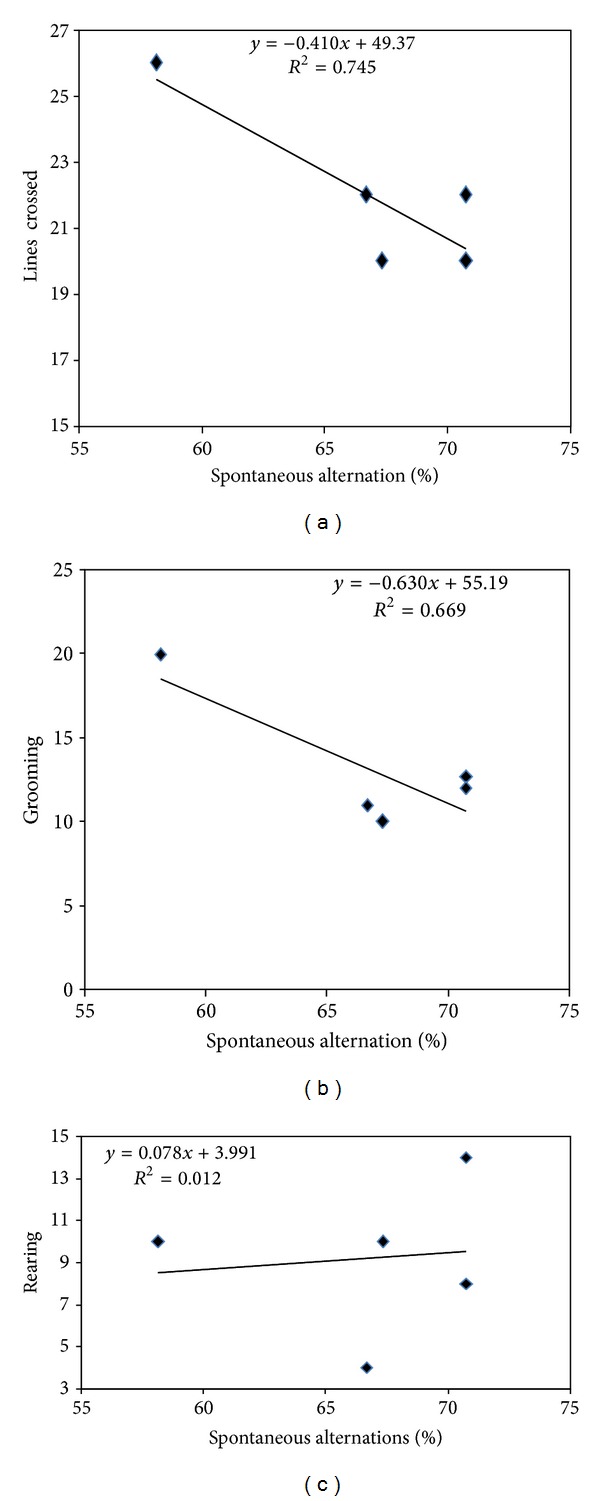
Correlation between working memory errors versus locomotion (a), grooming (b), and rearing (c) in the Y-maze activities of mice treated with the *T. dodoneifolius* at the dose of 50 mg/kg.

When linear regression was determined, negative correlation between spontaneous alternation versus number of entries in the maze (*n* = 5, *r* ^2^ = 0.745, *P* = 0.2678) (Figure 8(a)), between spontaneous alternation versus grooming time (*n* = 5, *r* ^2^ = 0.716, *P* = 0.358) (Figure 8(b)) were observed. However a positive but very low and therefore not significant correlation between spontaneous alternation versus number of rearing in the maze (*n* = 7, *r* ^2^ = 0.237, *P* = 0.2678) was noted (Figure 8(c)).

## 4. Discussion

In the present study, the anxiolytic, and antidepressant-like effects of the acute administration of TAE were studied in different animal models of anxiety and depression. The effect of this extract on memory improvement was later investigated using Y-maze paradigm.

The plant extract was first studied using the elevated plus maze which is considered to be an etiologically valid animal model of anxiety because it makes use of natural stimuli [23]. Anxiety-like behavior may be represented by an avoidance of the open arm and immobility of an animal placed in the elevated plus maze. In accordance with previously published reports, anxiolytic substances like diazepam increased the percentage of open arm entries and the time spent by animals in the open arms [24–26]. In this work, treatment of mice with TAE lead to a dose-dependent and significant (*P* < 0.01) decrease in the number of entries in closed arms. The time spent in open arms increased but was significant only at 100 mg/kg (*P* < 0.05). Diazepam, a benzodiazepine anxiolytic drug used as standard drug, also significantly (*P* < 0.001) increased the open arm exploration. It is quite important to notice that in the EPM, some stress parameters like rearing, grooming, and head dipping were significantly reduced when the animals were pretreated with the extract (*P* < 0.05) or with diazepam (*P* < 0.01), when compared to control mice. The increase in time spent in the open arms in the EPM is an indicator of reduction of anxiety in rodents [27]. However, in this study, the reduction of the time spent in the close arm was not systematically followed by an increase of the time in the open arm. The anxiolytic effects of benzodiazepine drugs are usually accompanied by decreased ambulatory activity and sedation. Taken together, these results highly suggested that the action of TAE may be more sedative than anxiolytic.

The OFT was also used for the investigation of the anxiolytic state of the mice. General activity in the OFT measures various behavioral parameters, among those related to emotional, exploratory, and motor behaviors. The first exposure of the animal to the open field has a more marked emotional component than the remaining aspects of exposure [28]. The results obtained with this animal model showed that diazepam (2 mg/kg), TAE at all the doses used, significantly decreased the number of lines crossed by mice (ambulatory activity). Moreover, the time spent at the center was also slightly increased. This may indicate that the plant possesses substances that may have sedative or anxiolytic properties. On the other hand, the reduced locomotion pattern of the mice in the OFT confirmed the result obtained with the EPM and strongly suggested that this plant may produce sedative effects [29]. It is well known that benzodiazepines have sedative and ataxic side effects [25, 30, 31]. This was entirely confirmed in our study, using 2 mg/kg of diazepam. However, it is noteworthy that a reduction in spontaneous activity can be due to a variety of causes other than sedation, such as motor impairment or muscle relaxation. Therefore, other behavioral evaluation, such as prolongation of pentobarbital-induced sleep are needed to confirm the sedative effect of TAE. Moreover since in the forced swimming test the result of the extract was an increase in swimming time, we tend to exclude the muscle relaxation amongst the causes of the reduction of spontaneous locomotion of mice. The remaining causes could thus be the sedation or the motor impairment. These findings raised the possibility that the anxiolytic or sedative and other central depressant effects of the extract may be exerted by different phytoconstituents possibly acting through different receptor subtypes or having different affinity for the relevant receptors. Further studies using different fractions of *T. dodoneifolius* will be addressed for these possibilities. Nevertheless, the reduction of anxiety in the two animal models used in our study may either be related to the action of the TAE on benzodiazepine sites of GABA_A_ receptor complex.

The TAE may bind to the gamma subunit of GABA-A receptor, increasing the GABA activity in the brain, through an increase in chloride ion conductance and inhibition of the action potential. This proposed mechanism may explain the powerful sedation and the anxiolytic effect of the extract as it is well known that benzodiazepines have this pharmacological profile. However an antagonist on 5-HT_1–3_ receptors can also be hypothesis [32, 33]. These anxiolytic effects may be related to their flavonoids content. Flavonoids with anxiolytic activity have been described in many plant species used in folk medicine such as *Passiflora caerulea* [34]. The TLC phytochemistry of *T. dodoneifolius* carried out by Wahab et al. [35] revealed the presence of terpenoids and terpenoid-related compounds. These substances have proven their efficiency in animal models of anxiety, pain, depression, and epilepsy [36].

The forced swimming test is a well-known behavioral test in rodents that predicts the clinical efficacy of many types of antidepressants and investigates the mechanism underlying their action [37]. Various antidepressant medications including serotonin reuptake inhibitors (SSRI), tricyclic antidepressants, or NMDA receptor antagonists reverse the immobility posture and promote the occurrence of escape-related behavior [38]. The immobility displayed by rodents when subjected to unavoidable stress such as forced swimming is thought to reflect a state of despair or lowered mood, which are thought to reflect depressive disorders in humans. Rodents when forced to swim in a cylinder from which they cannot escape will, after an initial period of vigorous activity, display a characteristic immobile posture which can be readily identified and is said to reflect a state of despair. Moreover, it has some drawbacks represented by the possibility of obtaining some false positive or negative responses [39]. Drugs that enhance motor activity may give a “false” positive effect in the forced swimming test. Therefore, this test would not be as a good test for antidepressants such as nomifensine, amineptine, and any plant extract exhibiting myostimulant activities, since these agents increase motor activity [39]. In this study, the single administration of 100 mg/kg of TAE provoked significant reduction of the immobility time of mice subjected to forced swimming as compared to the control group. The result was quite comparable to that of imipramine, the tricyclic antidepressant drug used as standard at the same dose of 10 mg/kg, indicating that the extract possesses antidepressant activity on the central nervous system.

To discount the possibility of false positive response, TAE was evaluated for its effects on spontaneous motor activity. As shown in the elevated plus maze and in the open field tests, the extract did not increase spontaneous motor activity in mice but had a significant inhibition on the locomotory activity. Putting these results together, we can strongly suggest that the antidepressant activity of the TAE has no link with any skeletal muscle stimulation. It is of interest to note that several established antidepressants decrease locomotory activity [40]. Depression is commonly accepted to be a disorder due to disturbances in neurotransmitters function, particularly serotonin, noradrenalin, and dopamine. Reduction in brain serotonin has been reported to be one of the most important etiological factors for genesis of depression, and the most widely used antidepressants namely SSRIs, increase extracellular availability of serotonin [18, 41, 42]. Thus TAE may exert its antidepressant effect through one or some of these central nervous neurotransmitters acting on glutaminergic receptors, GABAergic receptor, or serotonergic pathways. At this level, it is not possible to give the exact mechanism of action by which *T. dodoneifolius* acts. However we can hypothesize according to our results that the antidepressant effect of TAE appears to be related to enhancement of central noradrenergic and/or serotonergic neurotransmissions.

The Y-maze apparatus is useful for assessment of memory with little effort [43]. This type of maze tests has been used to assess the spatial learning behavior of mice by many scientists [44, 45]. It is also used to determine the effect of different drugs on learning and memory process. It is known worldwide that rodents particularly have exploratory behavior in the maze and this ability comes from their evolutionary history. In fact rodents are small burrowing animals that have spent millennia digging and finding their way around underground tunnels. In this behavioral paradigm, the spontaneous alternation behavior recorded is considered to reflect spatial working memory, which in turn is a form of short-term memory [46]. In the present study, animals treated with a single dose (50 and 100 mg/kg) of TAE showed an improvement of the memory compared to control group. However, this improvement was significant only at the dose of 50 mg/kg. It is important to note that the process of memory is really very complex and in our study, the plant extract was acutely given once. It will thus be important and maybe interesting to know the pattern of the results of this test if the extract is given chronically for one or two weeks.

In summary, the current work demonstrated that acute administration of the TAE produced anxiolytic and antidepressant effects with a sedative side effect on mice and it slightly improves short-term memory. We speculated that the antidepressant effect of TAE observed is not due to the inhibition of the locomotory activity. Although the action mechanisms of this extract still to be studied, our results bring the pharmacological evidence of the traditional use of this plant for the treatment of some neurological disorders.
